# Molecular clocks indicate turnover and diversification of modern coleoid cephalopods during the Mesozoic Marine Revolution

**DOI:** 10.1098/rspb.2016.2818

**Published:** 2017-03-15

**Authors:** Alastair R. Tanner, Dirk Fuchs, Inger E. Winkelmann, M. Thomas P. Gilbert, M. Sabrina Pankey, Ângela M. Ribeiro, Kevin M. Kocot, Kenneth M. Halanych, Todd H. Oakley, Rute R. da Fonseca, Davide Pisani, Jakob Vinther

**Affiliations:** 1 School of Biological Sciences, University of Bristol, Life Sciences Building, 24 Tyndall Avenue, Bristol BS8 1TQ, UK; 2 School of Earth Sciences, University of Bristol, Life Sciences Building, 24 Tyndall Avenue, Bristol BS8 1TQ, UK; 3 Earth and Planetary System Science, Department of Natural History Sciences, Hokkaido University, Sapporo, Japan; 4 Natural History Museum of Denmark, Øster Voldgade 5-7, 1350 Copenhagen, Denmark; 5 Trace and Environmental DNA Laboratory, Department of Environment and Agriculture, Curtin University, Perth, Western Australia, Australia; 6 NTNU University Museum, Norwegian University of Science and Technology, Trondheim, Norway; 7 Molecular, Cellular and Biomedical Sciences, University of New Hampshire, Durham, NH 03824, USA; 8 Department of Biology, University of Copenhagen, Ole Maaløes Vej 5, 2200 Copenhagen N, Denmark; 9 Department of Biological Sciences, University of Alabama, Box 870344, Tuscaloosa, AL 35487, USA; 10 Department of Biological Sciences, Auburn University, Auburn, AL 36830, USA; 11 Department of Ecology, Evolution, and Marine Biology, University of California, Santa Barbara, CA 93106, USA

**Keywords:** Cephalopoda, molecular phylogenetics, phylogenomics, molecular clocks

## Abstract

Coleoid cephalopod molluscs comprise squid, cuttlefish and octopuses, and represent nearly the entire diversity of modern cephalopods. Sophisticated adaptations such as the use of colour for camouflage and communication, jet propulsion and the ink sac highlight the unique nature of the group. Despite these striking adaptations, there are clear parallels in ecology between coleoids and bony fishes. The coleoid fossil record is limited, however, hindering confident analysis of the tempo and pattern of their evolution. Here we use a molecular dataset (180 genes, approx. 36 000 amino acids) of 26 cephalopod species to explore the phylogeny and timing of cephalopod evolution. We show that crown cephalopods diverged in the Silurian–Devonian, while crown coleoids had origins in the latest Palaeozoic. While the deep-sea vampire squid and dumbo octopuses have ancient origins extending to the Early Mesozoic Era, 242 ± 38 Ma, incirrate octopuses and the decabrachian coleoids (10-armed squid) diversified in the Jurassic Period. These divergence estimates highlight the modern diversity of coleoid cephalopods emerging in the Mesozoic Marine Revolution, a period that also witnessed the radiation of most ray-finned fish groups in addition to several other marine vertebrates. This suggests that that the origin of modern cephalopod biodiversity was contingent on ecological competition with marine vertebrates.

## 1. Introduction

Octopus, cuttlefish and squid showcase advanced intelligence, a wide range of body sizes, sophisticated camouflage and mimicry, unique jet-locomotion and ingenious decoy countermeasures in the ink sac [1–3]. Charismatic in these ways, and owing to their importance as fishing stocks, cephalopods have garnered great interest from ecologists and evolutionary biologists. However, cephalopod evolutionary relationships and divergence times have remained unclear, in part, owing to uncertainties in their fossil record. The past 540 Ma of cephalopod evolution can be viewed as having three ecologically distinct phases. Originally shelled, sea-floor-dwelling molluscs, cephalopods are descended from superficially limpet-like ancestors in the Cambrian [4,5]. The protective shell later became adapted as a chambered buoyancy organ [6], giving rise to free-swimming forms by the latest Cambrian that radiated into several Ordovician lineages [7]. Subsequently, internalization and reduction of the mineralized shell facilitated adaptation for alternative ecologies in the coleoids [8].

Anatomical evolution is in part shaped by the ecological relationships between predator– and prey species. Cephalopods (and in particular oceanic squid) fill a niche that largely overlaps with fishes as active mesopredators [9]. Considering the evolutionary trajectory of cephalopods from heavily shelled animals to rapid hunters, the question of how and when this development took place remains unresolved. Previously, coevolution between marine predators and prey has been hypothesized from the fossil record of the Jurassic and the Cretaceous, and this ecological shift has since become known as the Mesozoic Marine Revolution [10,11].

By contrast, the fossil record leaves limited insight on the providence of modern coleoid groups [12], despite their well-documented ancestors and relatives especially among the ammonites and belemnites. Their mineralized, chambered portion of the shell (phragmocone and rostrum) has a high potential for preservation, but as the phragmocone became internalized, reduced, and in many cases lost entirely, so too was a clear narrative through fossils. Soft tissue fossilization is rare, but cirrate and incirrate octopods are known from the Late Cretaceous (Cenomanian) Hâkel and Hâdjoula Lagerstätte, while cirrate forms and stem octobrachians are recorded in the Jurassic [13]; these are known to preserve the unmineralized gladius and soft tissues. Stem group decabrachians, such as belemnites and other belemnoids are known, preserving their phragmocones and, occasionally, soft tissues [14,15]. By contrast, the extant octopuses, cuttlefish and squid are characterized by shell reduction and loss [16], and are prone to major taphonomic biases in tissue preservation [14]. Consequently, clarifying evolution of coleoids from the Mid-Palaeozoic to the present must, therefore, rely on alternative palaeobiological approaches, such as the estimation of molecular divergence times.

The first molecular divergence times of cephalopod evolution recovered very ancient divergences for the coleoids [17], suggesting extensive gaps in the fossil record. However, these studies used controversial calibrations from the Late Palaeozoic, such as *Shimanskya* [18] and *Pohlsepia* [19], for which the assignment to the coleoid crown group is dubious [20]. Subsequent studies attempted to estimate cephalopod divergences using calibrations from outgroups, such as bivalves and gastropods and recovered much younger divergence estimates, that were surprisingly congruent, irrespective of differences both in methodology and gene sampling [20,21]. These independent studies recovered a divergence between the nautilids and the coleoids around the Silurian–Devonian boundary, or the earliest Devonian (approx. 415 Ma), which is congruent with unequivocal evidence for fossil stem group coleoids (ammonoids and bactritids) [22,23] and stem group nautilids [24] in the Early Devonian. Cephalopod beaks also appear in the fossil record in the Devonian [25]. These observations suggest that the fossil record documents the origin of the crown group and that the concomitant evolution of the beak [20] coincides with a dramatic shift in predator–prey dynamics, termed the Devonian Nekton Revolution [26]. The jawed vertebrates radiated at this time, incident with a global shift in predatory style towards increased high-metabolism predation and durophagy [27]. The coincidence of jawed vertebrates and beaked cephalopods radiating at the Silurian–Devonian boundary may thus be interpreted as a response to the changes in the predator–prey landscape.

To explore the tempo and mode of coleoid evolution, we assembled a dataset of 180 nuclear genes of consistent rate of molecular evolution, representing crown diversity across Coleoidea. Phylogenetic and molecular divergence time analyses were carried out in a Bayesian framework, applying a molecular evolution model accommodating rate and compositional heterogeneity.

## 2. Experimental procedures

For full details of experimental procedures, see the electronic supplementary material. We compiled a supermatrix with data from 56 species (electronic supplementary material, table S2) for 180 genes. Phylogeny was inferred from this superalignment using the software package PhyloBayes MPI v. 1.5a [28] under CAT + GTR + *Γ*. The maximum-likelihood software RAxML MPI v. 8.1.15 [29] was applied to the same dataset as used in Bayesian inference, applying LG + I + *Γ*.

PhyloBayes 3.3f was used to infer molecular divergence times under the CIR [30] clock model, soft-bounds of 0.05 and a Yule-process birth–death model, with topology fixed to that inferred by PhyloBayes MPI v. 1.5a. A prior was applied to the root of 565 ± 10 Ma, representing the root of lophotrochozoa. Eleven fossil calibration points were applied to the analysis, as shown in table (electronic supplementary material, table S1).

## 3. Results

**Figure 1.**
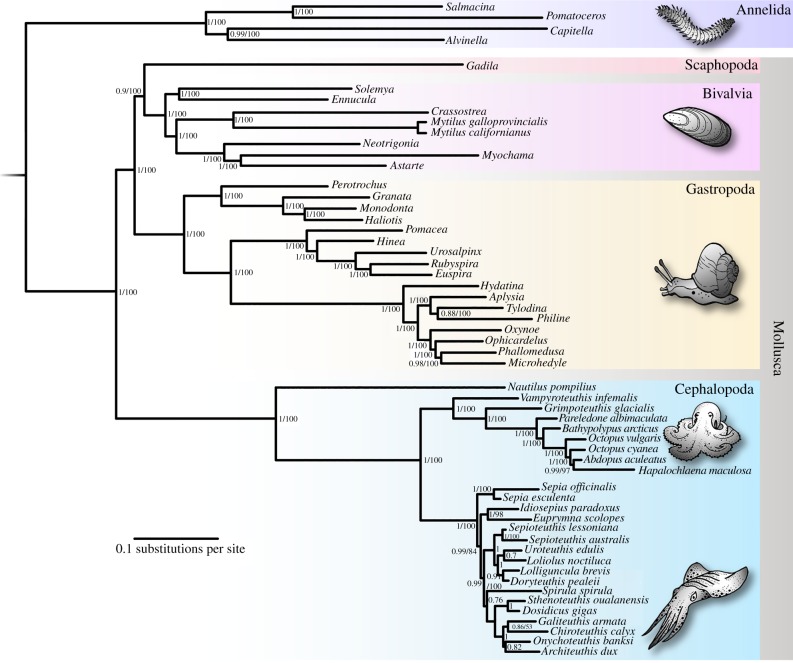
Molecular phylogeny of cephalopod, gastropod and bivalve molluscs (plus a scaphopod), with annelid outgroup; 180 genes, concatenated as 36 156 aligned amino acid positions with 26% missing data, modelled under CAT + GTR + *Γ*. Numbers at nodes denote Bayesian posterior probability/bootstrap support as returned by RAxML under the LG [33] substitution model. Scale bar is expected substitutions per site.

Our phylogenetic results confirm *Nautilus* as sister group to coleoids [20,31]. In turn, coleoids comprise two monophyletic groups: Octobrachia (Vampire squid, dumbo octopuses and incirrate octopuses) and Decabrachia (cuttlefish and squid, including *Spirula*), in agreement with morphology and previous molecular studies [16,17,32] (figure 1). The vampire squid *Vampyroteuthis* and the cirroctopod *Grimpoteuthis* represent cirrate octopuses, branching deep as successive sister groups to the incirrate octopuses (figure 1). Within Decabrachia, we recover a monophyletic Myopsida assemblage, along with support for Teuthoidea with the inclusion of *Spirula*, similar to previous studies [16,20]. However, the relationships between the orders comprising the Sepioidea (Sepiida, Idiosepiidae, Sepiolidae) are recovered as paraphyletic. Oegopsid monophyly is supported, with *Spirula* sister to this clade, in agreement with previous studies [16], but the posterior probability values for many decabrachian basal nodes are generally lower than in other parts of the phylogeny. Sepioid and myopsid relationships have proved difficult to resolve [16], and further phylogenetic work remains to clarify these.

**Figure 2.**
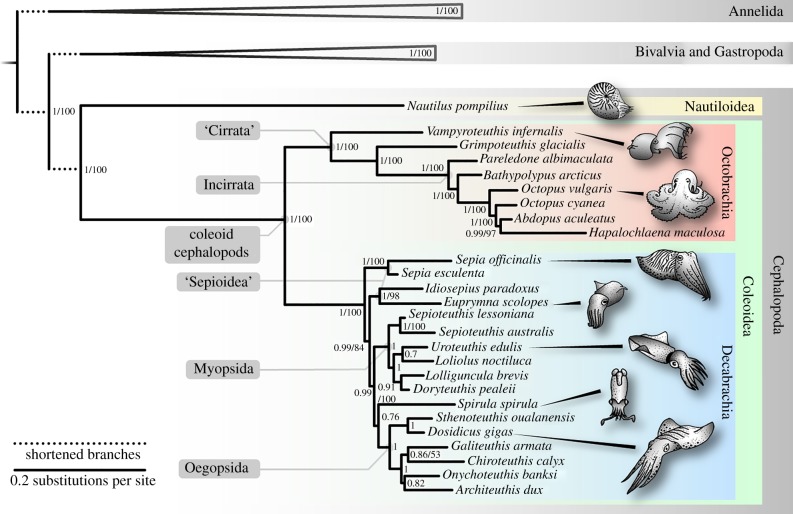
Phylogeny of 26 cephalopod species, plus outgroups (further details in figure 1); 180 genes, concatenated as 36 156 aligned amino acid positions with 26% missing data, modelled under CAT + GTR + *Γ*. Numbers at nodes denote Bayesian posterior probability/bootstrap support as returned by RAxML under the LG [33] substitution mode. Dotted branches at base of phylogeny are shortened for clarity, and outgroups (26 gastropods and bivalves, one scaphopod, four annelids) are collapsed for clarity (figure 1). Scale bar is expected substitutions per site.

**Figure 3.**
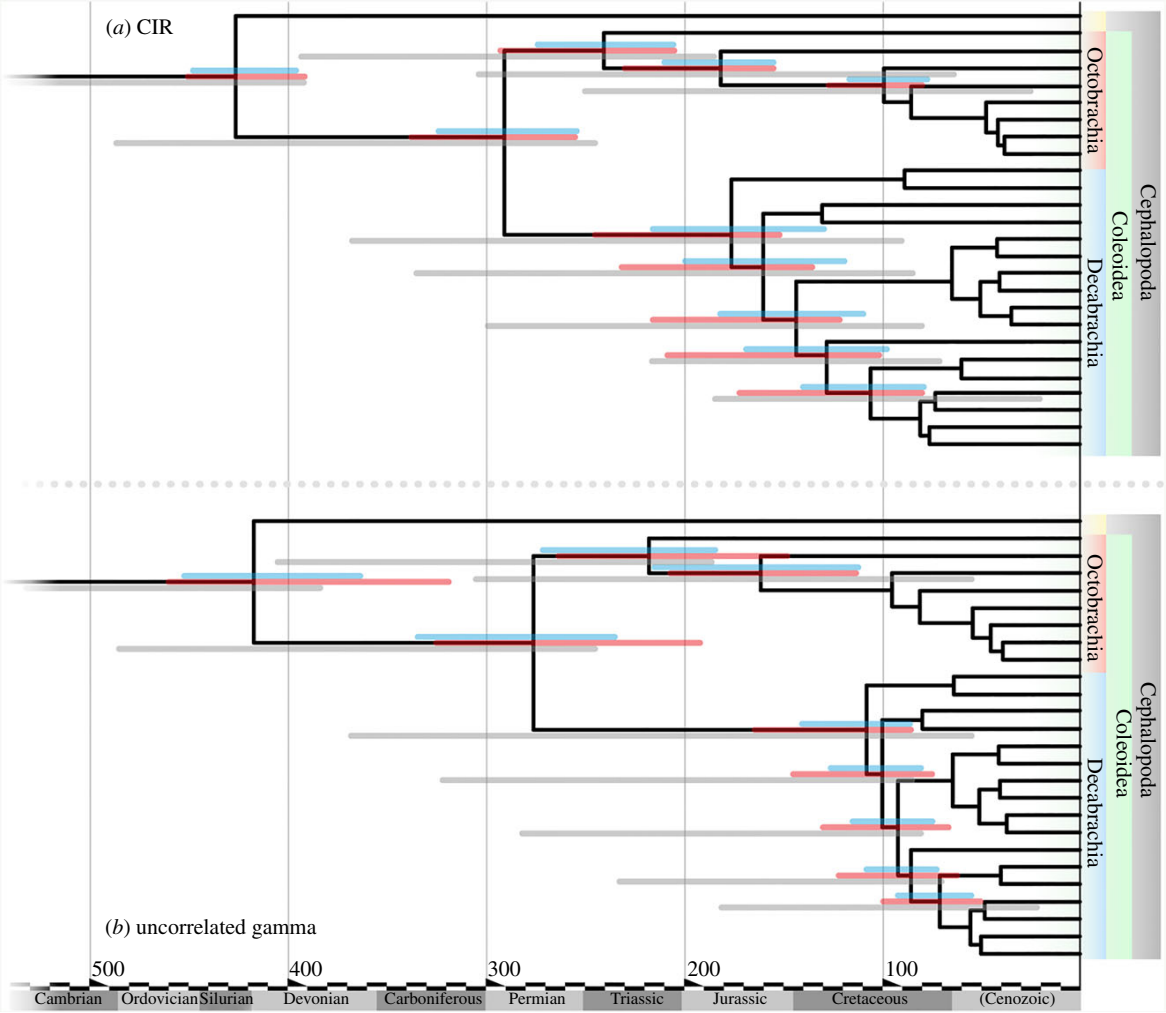
Comparison of molecular clock model and calibration scheme on confidence intervals for node timing inference. (*a*) Applying CIR clock model, (*b*) applying uncorrelated gamma multipier model. Red bars at nodes are confidence intervals with only calibrations external to cephalopods applied. Blue bars are confidence intervals with the full calibration applied. Grey bars are the joint prior distribution at nodes. Not all nodes are labelled to aid clarity, full details in the electronic supplementary material.

Molecular divergence times were estimated, from the same matrix used for phylogenetic inference, applying an autocorrelated relaxed clock model (CIR process, figures 2 and 3; electronic supplementary material for further details and additional analyses). Alternative treatments, model applications and comparison of the joint priors induced by our calibrations and models and the posterior divergence times supported the data as informative, and resulted in consistency in divergence time inference (figure 3; electronic supplementary material, table S3 and figure S3). Notably, our molecular divergence times are highly congruent with previous molecular divergence estimates [20,34] that used comparable calibration schemes. These studies, however, had insufficient taxonomic spread and sample required for more comprehensive investigation of the evolutionary tempo of coleoids. Furthermore, our wide sample represents crown diversity.

The oldest unequivocal crown group coleoids appear in the latest Triassic, with belemnites representing stem group decabrachians, and phragmoteuthidids (Early Triassic or latest Permian) proposed to represent stem group Octobrachia [35]. Our divergence times suggest that the coleoid crown diverged in the Late Carboniferous or Permian. Fossil consilience is shown by stem group vampire squid (loligosepiids) fossils of the earliest Jurassic (approx. 195 Ma) [13,36]. Octopus-like forms that are lacking the mantle fins and with reduced gladius appear in the latest Cretaceous (Cenomanian, 94–100 Ma) Lagerstätte of Hâkel and Hâdjoula, Lebanon [37]. Our divergence estimate for the incirrate octopods is in the Late Cretaceous (approx. 100 Ma). Decabrachians have a near non-existent fossil record, except for members of their stem group (e.g. belemnites) and some forms that retain remnants of the phragmocone—*Spirula* and cuttlefish. Stem group spirulids appear in the latest Cretaceous (approx. 66–72 Ma) of West Greenland [38]. Molecular estimates here suggest that spirulids diverged from the Oegopsids at approximately 128 Ma. Sepiid cuttlebones appear in the fossil record in the latest Cretaceous (approx. 75 Ma [37]) and we estimate the sepiids represented in our analysis to have diverged approximately 88 Ma.

## 4. Discussion

Our molecular divergence estimates show that the coleoid fossil record [13,39] belies not only an earlier origin for key cephalopod groups, but also significant differences in their rate of diversification. Together with the molecular clock estimates for coleoids that are lacking a fossil record, it is possible to investigate events that shaped the diversity of the group. Decabrachians diversify rapidly in the middle Mesozoic (Jurassic), while incirrate octopuses arose in the Cretaceous. Since this time documents an escalation—the evolution of novel predation strategies—it prompts a consideration of what anatomical changes took place in coleoids, particularly decabrachians, at this time.

The iconic shell has had a shifting functional role through cephalopod evolution, and is informative as to lifestyle and ecology. Subsequent to ancestral internalization of the phragmocone through the Carboniferous and Devonian, the decabrachian and octobrachian lineages independently evolved towards shell reduction [13,16], allowing enhanced manoeuvrability and speed [15]. These groups would have been in ecological competition with belemnites: stem group decabrachians [39,40] with an elaborate internal shell, diversifying in the Mid-Jurassic [41]. Our analysis suggests that in the Late Jurassic and at the onset of the Cretaceous, belemnites became marginalized and replaced by modern groups of decabrachians and finned octobrachians (figure 2) [13]. By retaining an elaborate internal phragmocone, belemnites could not compress their mantle cavity for jet propulsion to the same extent as the coleoid forms with a much more reduced internal shell. Similar patterns have been inferred from the Pacific fossil record in Japan [42], suggesting a dramatic turnover in particular approximately 100 Ma (figure 3).

**Figure 4.**
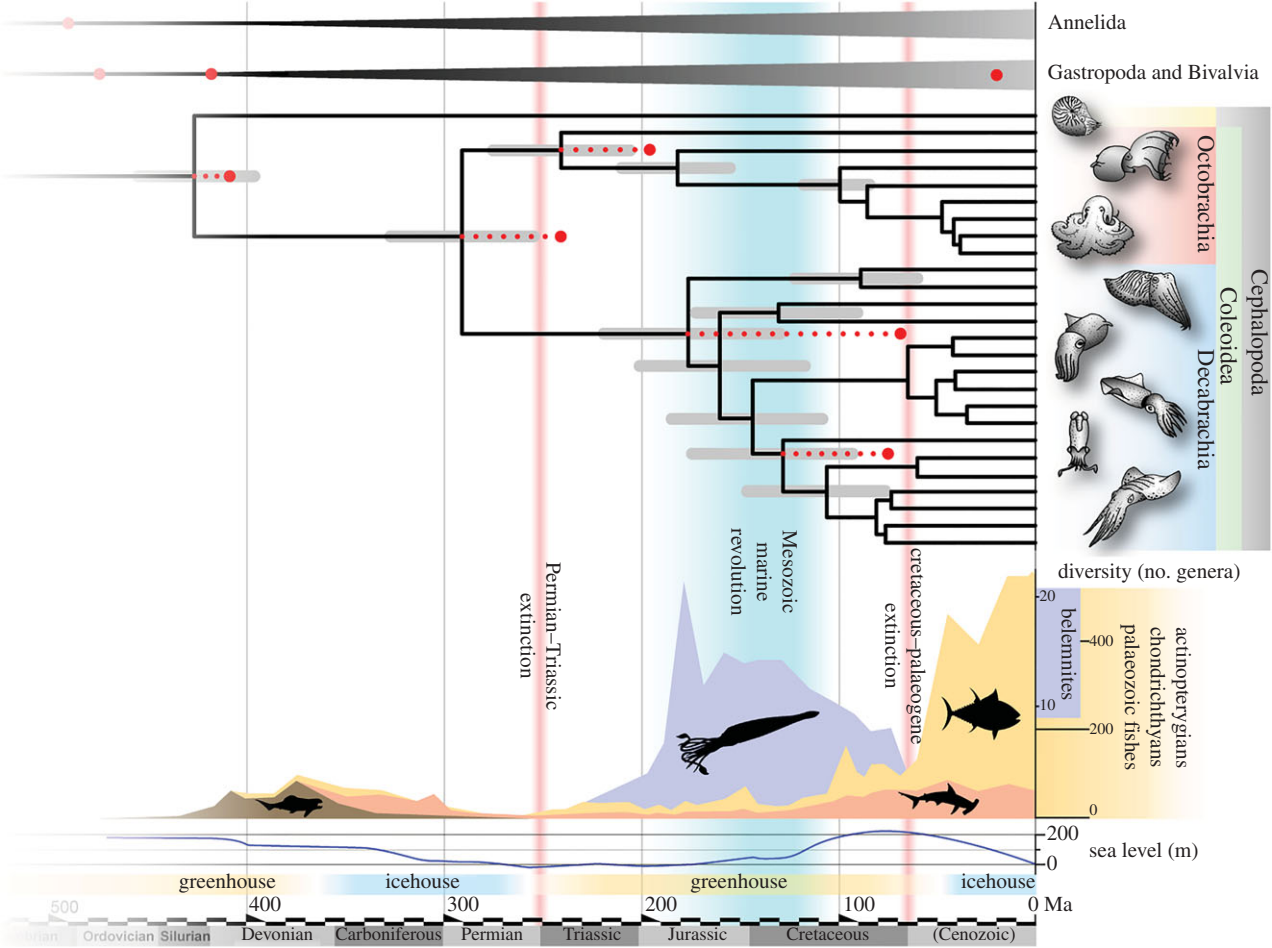
Chronogram of cephalopods, plus 26 bivalve and gastropod molluscs, one scaphopod and four annelids as outgroups and calibration nodes; 36 156 amino acid positions analysed under CAT-GTR substitution model, CIR clock model, Yule birth–death process, soft bound of 0.05, and a root prior of 565 Ma with a standard deviation of ±10 Ma. Bars at nodes represent 95% confidence intervals (recent nodes not labelled with bars to aid clarity). Red dots indicated calibrated nodes (electronic supplementary material, table S1 and figure S3); red dotted lines represent extent of calibration minima. Environmental conditions and sea-level curve simplified from Miller *et al.* [45]. Curves for belemnite, actinopterygian, chondrichthyan and Palaeozoic fish diversity are based on fossil observations on diversity, data from Palaeobiology Database (pbdb.org), electronic supplementary material, table S5. Red vertical lines represent major extinction events. Aqua-blue vertical bar signifies the extent of the Mesozoic Marine Revolution [10].

Decabrachian coleoids are nektonic predators with streamlined morphology, high metabolic rates and shoaling behaviour; adaptations in common with teleost fishes [43]. The majority of modern teleost groups radiated during the Jurassic and Cretaceous [44], concomitantly with the origin of most modern coleoids as revealed by our molecular estimates and the fossil record. The scenario in which Mesozoic ecological shifts are exhibited in teleost fishes, chondrichthyans (sharks and rays), and shelled invertebrates as investigated by Vermeij [10] can be extended to cephalopods (figure 4). In the face of high-metabolism, robust predators and niche-competitors, the cephalopods may have responded in kind to these evolutionary pressures. We hypothesize that the cephalopods evolved into the forms we are familiar with today, while shelled groups fell into extinction owing to the shifts in predation in this time period. The Mesozoic Marine Revolution can thus be viewed as the final stage in the shift from Palaeozoic ecologies into the modern structure of marine ecosystems, where (at least in the nektonic realm), agility superseded passive defence.

Ammonoids are stem group coleoids, which were common throughout the Late Palaeozoic until the end of the Mesozoic. Evidence from their radula morphology [23,46] suggests that ammonoids primitively had stout teeth, similar to macrophagous predatory cephalopods. In the Jurassic, the group evolved an enlarged calcareous lower jaw (aptychus) and longer, multicuspidate radula teeth, which has been attributed to a shift into microphagous suspension feeding [23,47]. As such, the group ‘stepped out’ of the arms race and ecological competition with the macrophagous predatory coleoids, fishes and marine reptiles during the Jurassic and Cretaceous. The group evolve increasingly ornamented shells in response to increased predation, as revealed from shell repair scar frequency [48], but eventually became extinct at the end of the Cretaceous.

## 5. Conclusion

Taken together, molecular divergence times and the cephalopod fossil record are consistent with a scenario in which predator–prey arms races shaped the coleoid body plan, biodiversity and ecology. The coincidence with the evolution of jawed vertebrates and teleost fishes during the Devonian Nekton Revolution and the Mesozoic Marine Revolution, suggests that nektonic marine vertebrates have been key antagonists towards cephalopods throughout most of their evolution.

## Supplementary Material

Further analyses, statistics, and data-acquisition details.

## Data accessibility

All sequence information archived on NCBI, see the electronic supplementary material for accessions. All matrices available on Dryad, doi:10.5061/dryad.180nh [49].
