# Supplementary material for: Molecular clocks indicate turnover and diversification of modern coleoid cephalopods during the Mesozoic Marine Revolution
Source: Proc Biol Sci. 2017 Mar 15;284(1850):20162818. doi: 10.1098/rspb.2016.2818 (PMC5360930; doi:10.1098/rspb.2016.2818)
Supplement: Further analyses, statistics, and data-acquisition details. [file rspb20162818supp1.pdf]

Supplementary information

Title
Molecular clocks indicate turnover and diversification of cephalopod molluscs during the Mesozoic Marine Revolution.

Authors
Tanner Alastair R., Dirk Fuchs, Winkelmann Inger E., Gilbert M. Thomas P., Sabrina Pankey, Ribeiro Ângela M., Kocot Kevin M., Halanych Kenneth M., Oakley Todd, da Fonseca Rute R., Pisani Davide and Vinther Jakob.

Experimental procedures

(a) Phylogenomic data assembly

We compiled a supermatrix with data from 56 species (see SI table 2) for 197 genes, based on the dataset of Philippe et al. [1]. This set was selected due to substitution rate consistency of the gene-sample, and since differences in read-depth of the transcriptomic data would lead to relatively sparse orthology groups. New cephalopod sequences matching those in the Philippe gene-sample were acquired through BLAST [2] searching transcriptomic sequences, using *Aplysia californica* as the search query due to this taxon possessing full coverage for the gene dataset, and having phylogenetic proximity to the group in question. A custom Perl script (available at github.com/jairly/MoSuMa_tools/) selected sequences on the most significant expect values (e-values) among BLAST hits, taking the lowest e-values and any other significant hits within three orders of magnitude of the most significant hit. The maximum e-value threshold was set at 10^{-10}, with hits exceeding this being excluded. These selected sequences were aligned using MUSCLE [3] (default parameters), to produce gene-alignments for each of the 197 genes (see SI table 3). Ambiguously aligned positions were removed from the gene alignments by GBlocks v0.91b [4] (*b2* = 70%, *b3* = 10, *b4* = 5, *b5* = half). The output of the GBlocks was concatenated using SequenceMatrix v100 [5], with a resulting supermatrix of 36,156 amino acid positions across 56 taxa.

(b) Phylogenetic inference

The superalignment was analysed using the Markov Chain Monte Carlo (MCMC) sampler PhyloBayes MPI v1.5a [6]. The mixture model CAT + GTR + Γ_4 was applied, being the most appropriate to deal with across-site heterogeneities, while minimising long-branch biases. Two independent Monte Carlo chains were run, a burn-in of 25% of the Markov chains were discarded. These chains converged, with the maximum difference in the bipartitions of the chains < 0.1, as reported by *bpcomp* program in the PhyloBayes package. A further test of convergence was carried out using *tracecomp* (also under PhyloBayes), with effective sample sizes being > 50, and relative differences dropping below 0.1 for all parameters as compared between the independent chains. The maximum likelihood software RAxML MPI v8.1.15 [7] was applied to the same dataset as used in Bayesian inference, applying LG + I + Γ_4. 1000 pseudoreplicates were run.

(c) Molecular divergence time inference

Phylobayes 3.3f was used to infer molecular divergence times using CAT + GTR, soft-bounds of 0.05, and a Yule-process birth-death model. A Bayes Factor analysis of the fit of three alternative models was performed (CIR [8], log-normal [9], and uncorrelated gamma [10]), with CIR showing a marginally better model-fit. Of these models, CIR was applied due to its ability to model rate change along branches and between taxa, while avoiding over-relaxation of divergence time inference.

The topology was fixed to that inferred by PhyloBayes MPI v1.5a, the root constrained to the bifurcation between the uncontroversial monophyletic assemblages of annelids and molluscs (cephalopoda + bivalvia + gastropoda + scaphopoda); the bivalves and gastropods, plus annelids were considered a balanced outgroup (with comparable taxonomic sampling and phylogenetic crown spread). A prior was applied to the root of 565 ± 10 Ma, representing the root of lophotrochozoa [11]. This prior was tested as being appropriate by chains being run without data, to confirm that the samples were being drawn from a distribution that includes the prior. The root age of the prior run was 552 ± 8 Ma, supporting the prior as appropriate.

Eleven fossil calibration points were applied to the analysis, as shown in table (SI table 1). Two independent MCMC chains were run for each model, with convergence being determined through *tracecomp*, with effective sample sizes > 50, and relative differences < 1 for all parameters as compared between independent chains. The discarded burn-in was 25% of the chain length.

Full phylogeny with outgroups

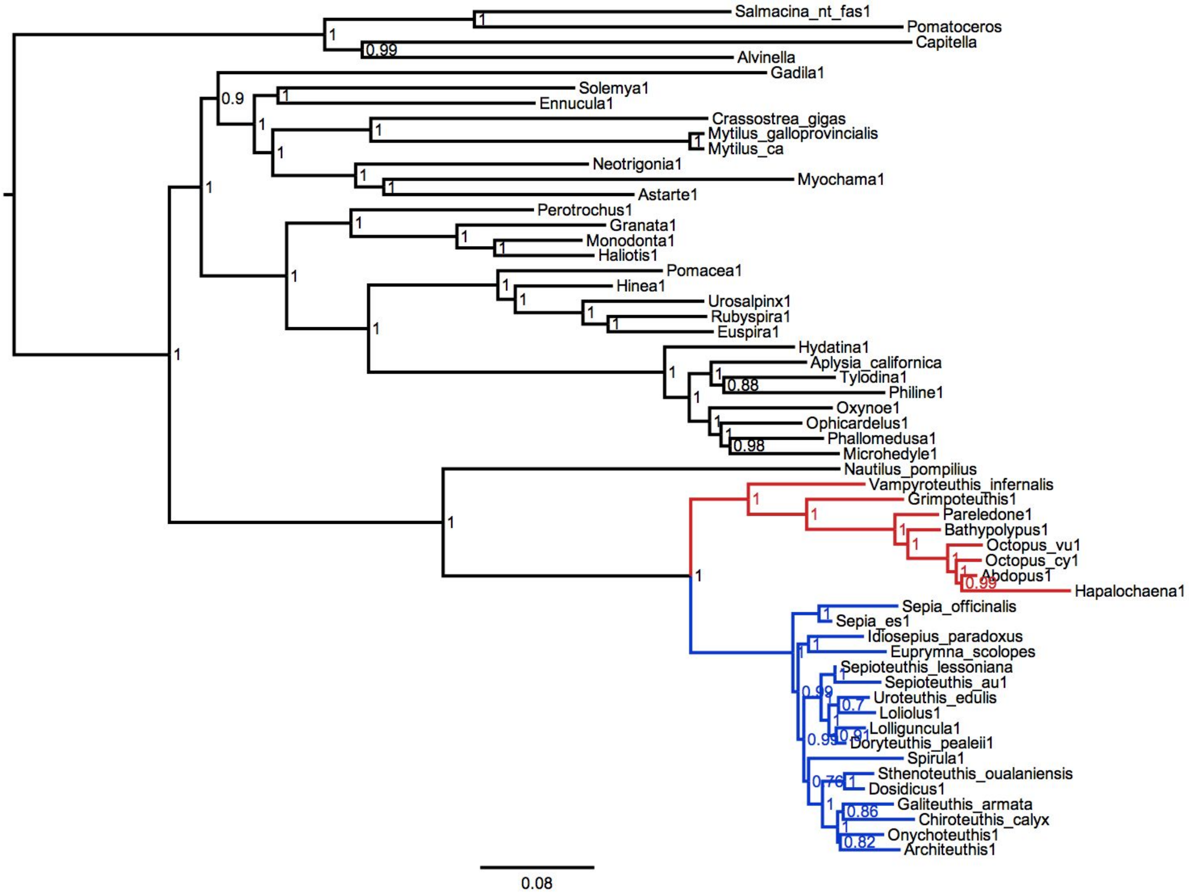

Supplementary figure 1: Molecular phylogeny of 26 cephalopod species, plus outgroups. 180 genes, concatenated as 36,156 aligned amino acid positions with 26% missing data, modelled under CAT + GTR + Γ. Numbers at nodes represent posterior probabilities.

Data acquisiton

Genomic data for *Architeuthis dux*, and transcriptomic data for *Onychoteuthis banksi* and *Sthenoteuthis banksi* were generated and assembled by the Sequencing Centre of the University of Copenhagen using the Qiagen RNeasy extraction protocol, and the TruSeq RNA Kit v2 RNA isolation, cDNA synthesis, ligation and PCR-amplification protocol. Quality control was carried out using a Bioanalyzer 2100, Agilent Technologies. Transcriptomic sequence data from *Bathypolypus arcticus, Grimpoteuthis glacialis, Lolliguncula brevis* and *Spirula spirula* were generated at the University of Bristol Life Sciences Sequencing Facility. For these species, the Trizol extraction protocol was applied, with sequencing carried out on the Illumina HiSeq platform, 100 base pair read length, paired end reading. Transcriptome assembly was carried out in Trinity version 2.0.3 [12] under default parameters and using Trimmomatic (default parameters, as part of the Trinity package) for quality control. See supplementary information for accession numbers of published sequence data.

Orthology assesment

Gene trees were assessed for orthology applying for following protocol. Maximum likelihood phylogenies were inferred for each gene using PhyML [13] version 3, modelling under LG

[14] and accepting the best tree of either SPR or NJ. Sequences producing long branches were removed from the alignments, with a long branch considered to be more than 2 times the standard deviation of the average away from the average branch length for the gene in question (script available at github.com/jairly/MoSuMa_tools/). 17 genes were considered to have low orthology confidence (due to unresolved gene trees) across the taxonomic sample, and discarded, leaving 180 gene alignments. Ambiguously aligned positions were removed from the gene alignments by GBlocks v0.91b [4] (*b2* = 70%, *b3* = 10, *b4* = 5, *b5* = half).

To provide alternative topological inference, maximum likelihood approaches were also employed. PartitionFinder [15] and IQTree [16] were used to test model fit under maximum likelihood, with both returning the substitution model of Le and Gascuel [14], with a gamma distribution of rates and a proportion of invariant sites as having best model fit.

Fossil calibrations

12 fossil calibrations were applied to the molecular clock analyses. Letters in supplementary table 2 below refer to the nodes labelled a-l in supplementary figure 2. The root itself was constrained to 565 ± 10 Ma, as evidenced by *Kimberella* of the Douoshanto [11,17]. The root prior was tested as appropriate by running the analysis without data, returning a posterior distribution on the root of mean 565 ± 9.8 Ma, supporting the priors as appropriate.

Node	Maximum	Minimum	Reference	Position
a: Scaphopoda + Bivalvia + Gastropoda	543 Ma	525 Ma	Benton *et al.* 2015 [18]	External

b: Scaphopoda + Bivalvia	-	532 Ma	Nutzel *et al.* 2000 [19]	External
c: Bivalvia	-	485 Ma	Parkhaev 2008 [20], pp. 33-69	External
d: Vetigastropoda	-	490 Ma	Edgecombe *et al.* 2011[6]	External
e: Caenogastropoda + Heterobranchia	-	418 Ma	Frýda *et al.* 2008 [21], pp. 239-270	External
f: *M. edulis* + *M. californianus*	-	20 Ma	Bacon *et al.* 2015	External
g: Crown Decabrachia	-	68 Ma	Klug et al. 2016	Internal
h: *Spirula*, plus descendents	-	75 Ma	Fuchs *et al.* 2013 [22]	Internal
i: Crown Vampyromorpha	-	195 Ma	Fuchs & Weis 2008 [23]	Internal
j: Crown Coleoidae	-	240 Ma	Kroger & Mapes 2007 [24]	Internal
k: Crown Cephalopoda	-	408 Ma	Kroger & Mapes 2007 [24]	Internal
l: Crown Mollusca	549 Ma	-	Grant *et al*. 1991 [25]	External

Supplementary table 1: fossil calibrations.

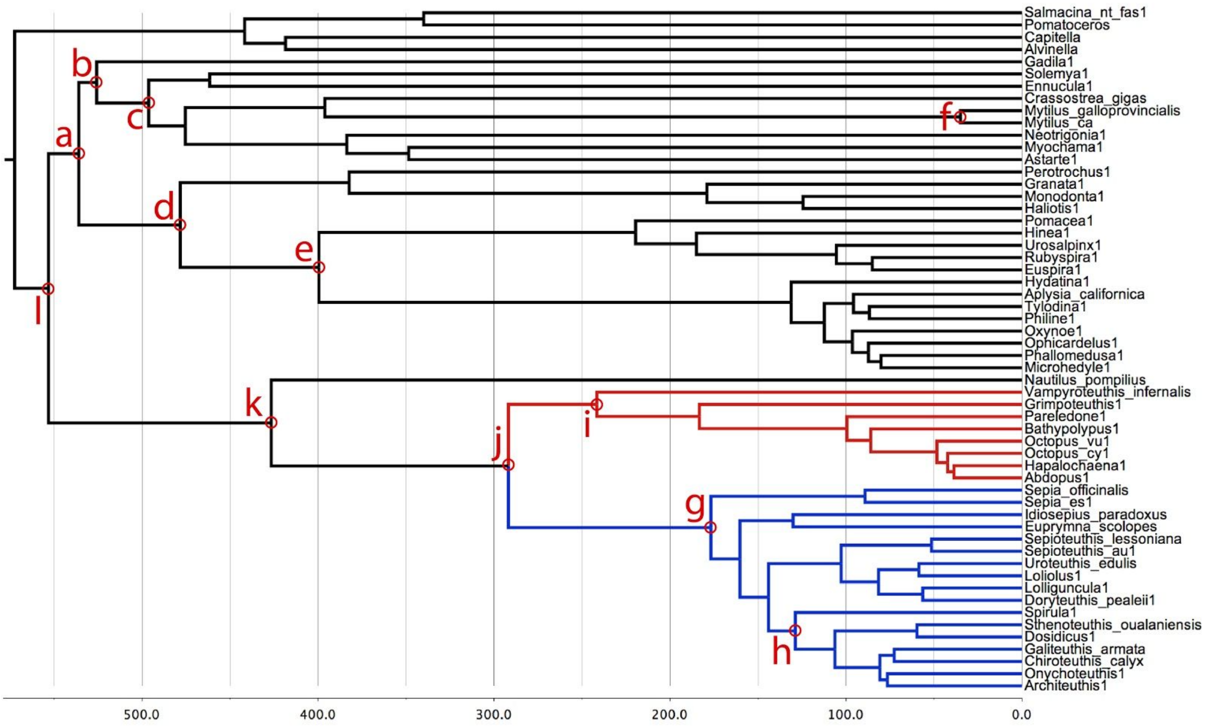

Supplementary figure 2: Calibration positions. 36,156 amino acid position, 56 taxa, CAT + GTR substitution model, CIR clock model, 12 calibrations (see SI table 1), root prior of 565 ± 10 Ma, soft bounds of 0.05, Yule birth-death process. Outgroup constrained to annelid monophyly.

Clock model cross-validation

Cross-validation was carried out in accordance with guidelines in the PhyloBayes manual, comparing the autocorrelated CIR model against the uncorrelated UGAM model. With CIR against UGAM, Bayes factor returned as 40.87 ± 165.7; a positive number is support for the fit of CIR.

Alternative clock models and calibration schemes

Analyses were run to assess the impact of internal versus external calibrations on divergence time estimations. See supplementary table 1 for calibrations classified as "internal" and "external". Note that "internal" refers to application of *both* internal and external calibrations.

Treatment	Crown Cephalopoda	Crown Coleoidae	Crown Octobrachia	Crown Decabrachia	Oegopsida	Myopsida	Incirrata
CIR externally calibrated	434	306	260	202	122	119	107
CIR internally calibrated	426	289	239	173	104	100	98
UGAM externally calibrated	405	264	210	123	74	65	101
UGAM internally calibrated	423	280	220	110	72	66	96

Supplementary table 2: Inferred divergence ages of key nodes under alternative clock model and calibration scheme.

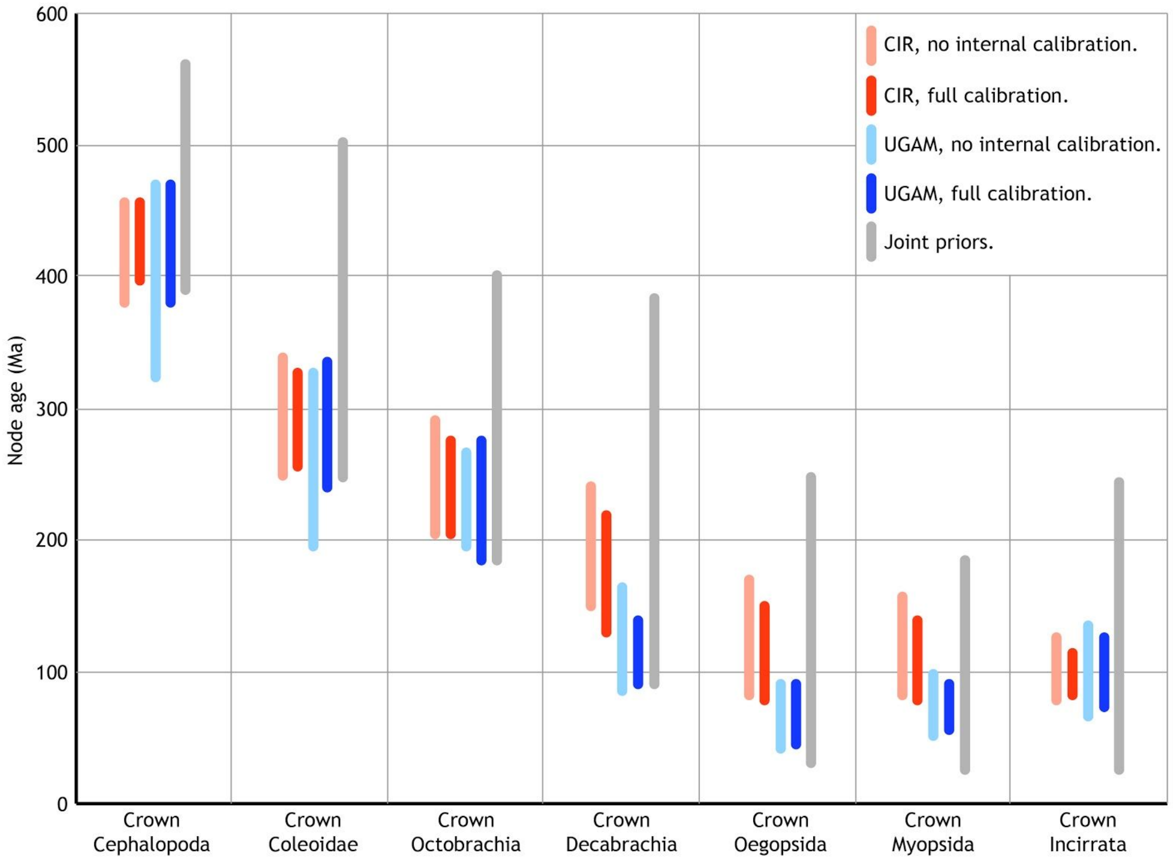

Supplementary figure 3: Inferred divergence ages of key nodes under alternative clock model and calibration scheme.

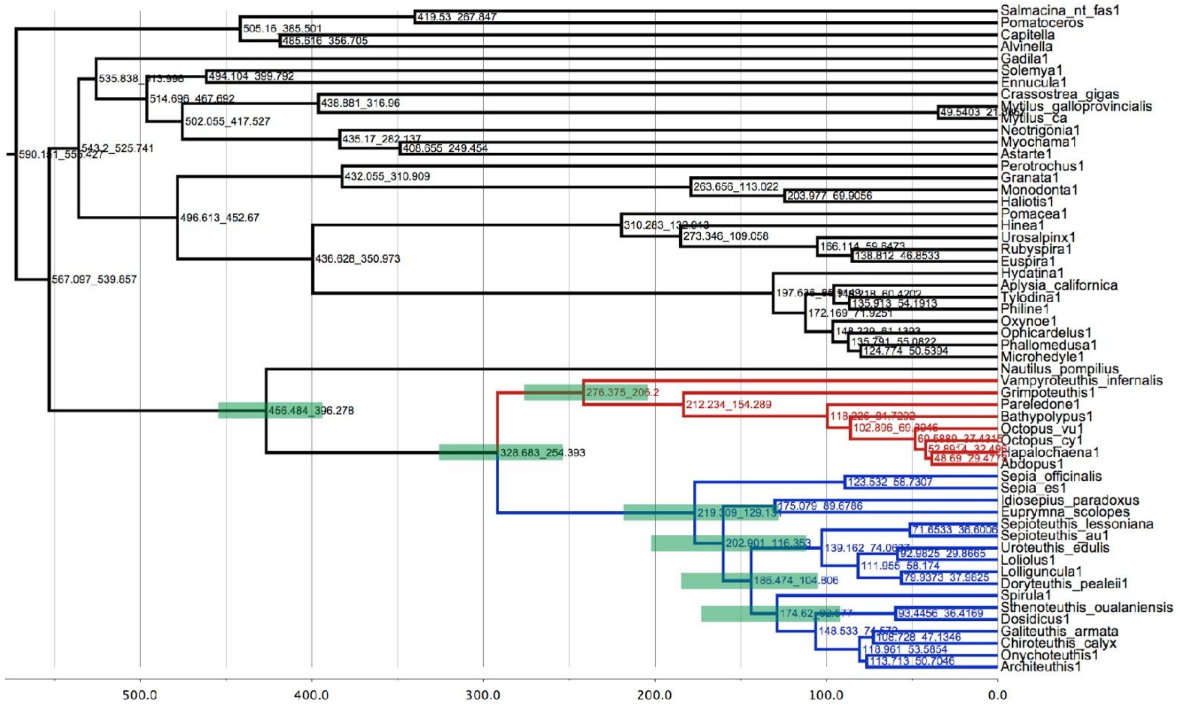

Supplementary figure 4: CIR clock. 36,156 amino acids, 52 taxa, CAT + GTR substitution model, CIR clock model, 12 calibrations, with annelid outgroup (see SI fig 2), root prior of 565 ± 10 Ma, soft bounds of 0.05, Yule birth-death process.

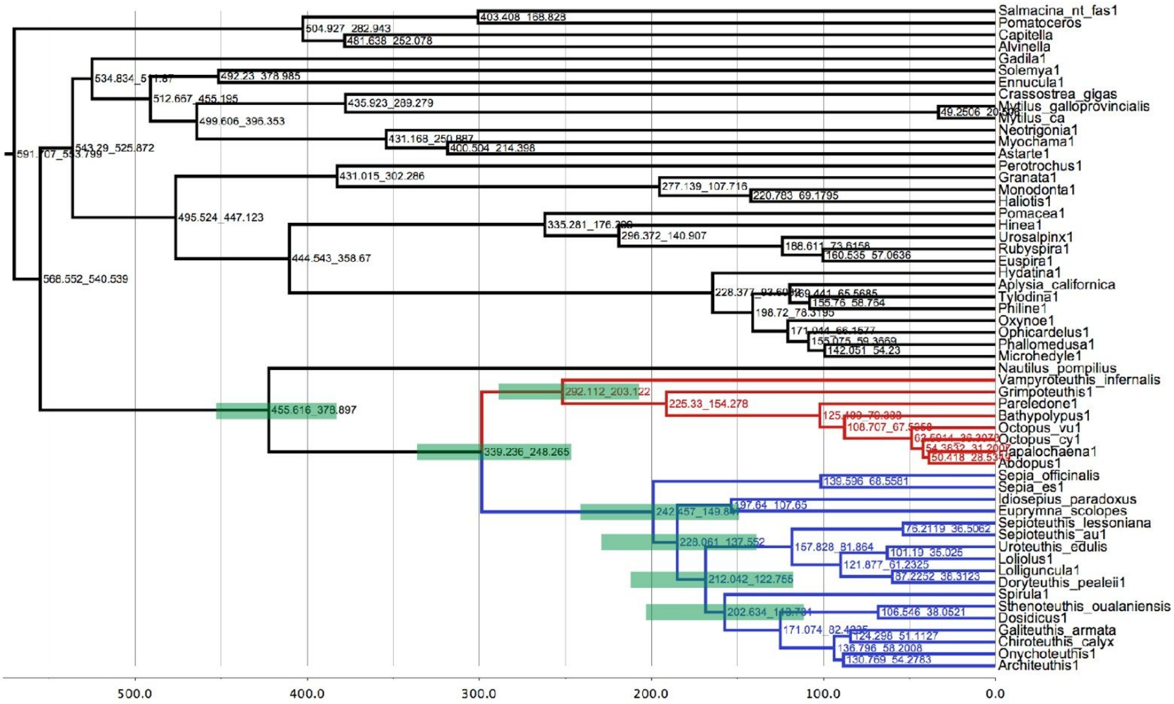

Supplementary figure 5: CIR clock. 36,156 amino acid position, 52 taxa, CAT + GTR substitution model, CIR clock model, 6 calibrations with none internal to Coleoidae, with annelid outgroup (see SI fig 2), root prior of 565 ± 10 Ma, soft bounds of 0.05, Yule birth-death process.

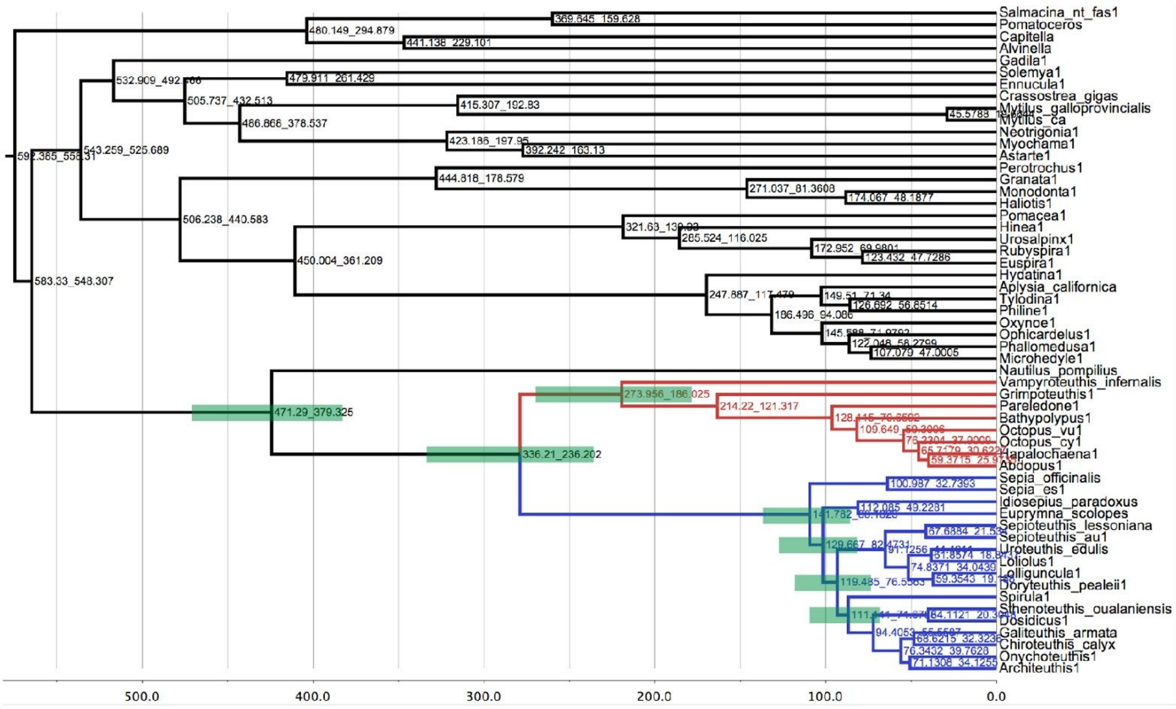

Supplementary figure 6: UGAM clock. 36,156 amino acids, 52 taxa, CAT + GTR substitution model, UGAM clock model, 12 calibrations, with annelid outgroup (see SI fig 2), root prior of 565 ± 10 Ma, soft bounds of 0.05, Yule birth-death process.

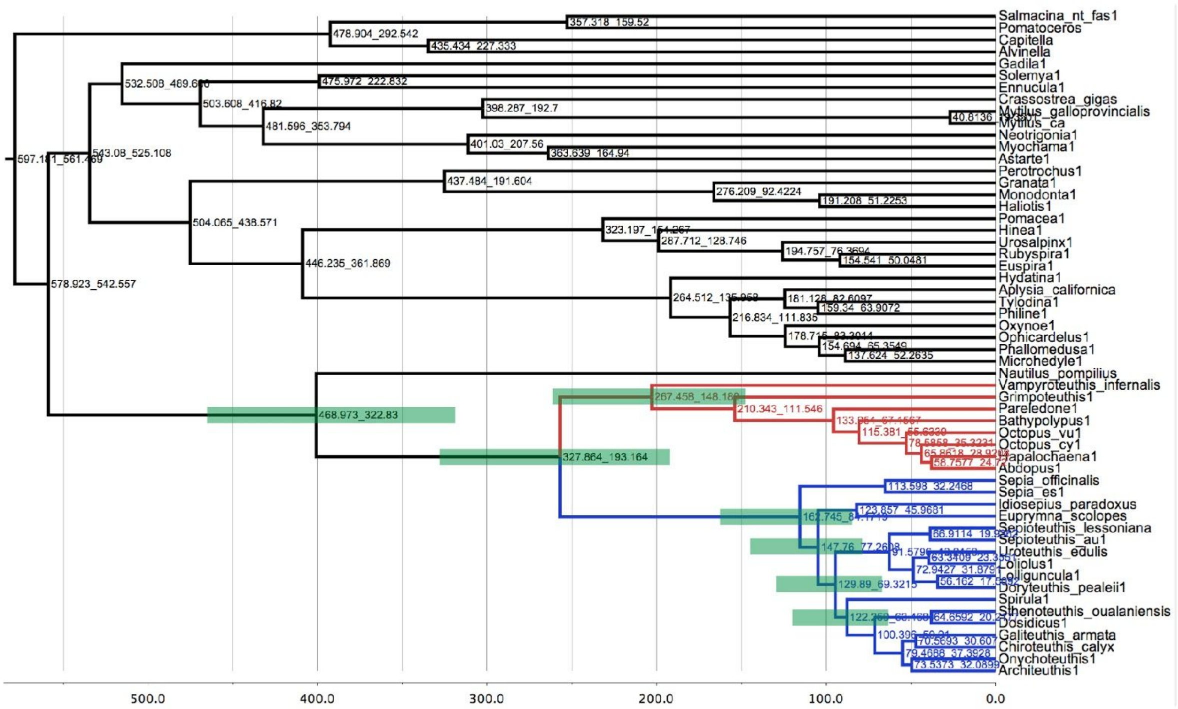

Supplementary figure 7: UGAM clock. 36,156 amino acids, 52 taxa, CAT + GTR substitution model, UGAM clock model, 6 calibrations with none internal to Coleoidae, with annelid outgroup (see SI fig 2), root prior of 565 ± 10 Ma, soft bounds of 0.05, Yule birth-death process.

Matrix completeness and NCBI accessions

Species	#genes	% Completeness	AA positions	Accession
Aplysia sp.	**180**	**100.00**	**36156**	**PRJNA253054**
Hydatina sp.	**180**	**100.00**	**36156**	**PRJNA253054**
Phallomedusa	**180**	**100.00**	**36156**	**PRJNA253054**
Oxynoe sp.	**179**	**99.44**	**36086**	**PRJNA253054**
Tylodina sp.	**179**	**99.44**	**35986**	**PRJNA253054**
Philine sp.	**179**	**99.44**	**35792**	**PRJNA253054**
Sepia esculenta	**176**	**97.78**	**35449**	**SRR1386223**
Monodonta sp.	**175**	**97.22**	**35406**	**PRJNA253054**
Urosalpinx sp.	**175**	**97.22**	**35099**	**PRJNA253054**
Sepia officinalis	**174**	**96.67**	**34854**	**SRR1325115**
Solemya sp.	**171**	**95.00**	**34583**	**PRJNA253054**
Rubyspira sp.	**172**	**95.56**	**34545**	**PRJNA253054**

Hinea sp.	171	95.00	34382	PRJNA253054
Neotrigonia sp.	170	94.44	34298	PRJNA253054
Ennucula sp.	168	93.33	34172	PRJNA253054
Microhedyle sp.	163	90.56	34153	PRJNA253054
Gadila sp.	165	91.67	33487	PRJNA253054
Euspira sp.	167	92.78	33326	PRJNA253054
Granata sp.	167	92.78	33098	PRJNA253054
Dosidicus sp.	153	85.00	32709	SRR1386212
Pareledone sp.	155	86.11	32484	SRR725936
Grimpoteuthis sp.	154	85.56	32382	[Matrix on dryad]
Bathypolypus sp.	153	85.00	32097	[Matrix on dryad]
Euprymna scolopes	157	87.22	31918	SRR3472306
Lolliguncula sp.	147	81.67	31474	[Matrix on dryad]
Sepioteuthis lessoniana	160	88.89	31438	SRR1386192
Pomacea sp.	154	85.56	31129	PRJNA253054
Doryteuthis sp.	140	77.78	30847	SRR3472305
Crassostrea sp.	146	81.11	30643	PRJNA253054
Octopus vulgaris	149	82.78	30588	SRR725937
Myochama sp.	147	81.67	29805	PRJNA253054
Sthenoteuthis sp.	133	73.89	28643	[Matrix on dryad]
Architeuthis sp.	133	73.89	28241	[Matrix on dryad]
Pomatoceros lamarkii	132	71.00	27003	SRR1810802
Mytilus californianus	120	66.67	26680	PRJNA253054
Haliotis sp.	137	76.11	25388	PRJNA253054
Onychoteuthis sp.	110	61.11	25094	[Matrix on dryad]
Perotrochus sp.	94	52.22	22666	PRJNA253054
Alvinella sp.	92	50.72	22144	[Matrix on dryad]
Capitella sp.	91	49.19	21711	SRR4045567
Idiosepius paradoxus	78	43.33	17112	SRR2984343

Chiroteuthis calyx	**81**	**45.00**	**15630**	**SRR2102319**
Sepioteuthis australis	**71**	**39.44**	**14835**	**SRR725780**
Salmacina sp.	**70**	**38.35**	**14635**	**[Matrix on dryad]**
Sepia officinalis	**60**	**33.33**	**14284**	**SRR1325115**
Abdopus aculeatus	**74**	**41.11**	**13398**	**SRR680047**
Uroteuthis sp.	**72**	**40.00**	**13083**	**DRR068682**
Mytilus edulis	**68**	**37.78**	**12849**	**PRJNA253054**
Octopus cyanea	**67**	**37.22**	**12314**	**SRR725937**
Galiteuthis armata	**57**	**31.67**	**11522**	**SRR2102359**
Nautilus pompilius	**36**	**20.00**	**8321**	**SRR108979**
Vampyroteuthis infernalis	**39**	**21.67**	**8218**	**SRR2102472**
Hapalochlaena maculosa	**30**	**16.67**	**6501**	**SRR3105559**
Spirula spirula	**25**	**13.89**	**6120**	**[Matrix on dryad]**
Loliolus noctiluca	**22**	**12.22**	**5177**	**SRR725597**

Supplementary table 3: data sources.

Palaeobiology Database queries

To generate diversity curves for vertebrates and belemnites in figure 1, occurrence data was retrieved from PBDB (pbdb.org). Queries can be repeated using the queries below, under default search options.

Query	Occurrences
**Belemnitida**	1565
**Actinopterygii**	6973
**Chondrichthyes**	10576
**Placodermi**	74
**Galeaspida**	26
**Osteostraci**	16
**Thelodonti**	78
**Anaspida**	10

Supplementary table 4: PBDB queries and number of records returned.

Alternative phylogenetic methods and key node placements.

Topology / Treatment	Idio/Euprym position	Sepiolida position	Architetuthis sister	Spirula placement
Original SM, metazoan OG, Dayhoff recoded	Sister of all Decabrachia / paraphyletic	Derived Myposid	Chiroteuthis	n/a
Original SM, metazoan OG, fast (1356 chars)	Sister of all Decabrachia / paraphyletic	Sister of all Deca, except Idio/Euprym	Onychoteuthis	Oegopsid sister
Original SM, metazoan OG, slow 36467 chars)	Myopsid sister	Myopsid root	Galiteuthis + Chiroteuthis	Polytomy at oegopsid root
Original SM, annelid OG, Spirula 100% 6412 chars	Sister of all Decabrachia	Sister of all Deca, except Idio/Euprym	Chiroteuthis	Oegopsid sister
Full matrix, gas/biv OG	Sister of all Deca, excpt Sepia	Sister of all Decabrachia	Onychoteuthis	Oegopsid sister
Ceph only, repeat gblocks ~15,000 chars	Sister of all Deca	Myopsid root	Onychoteuthis	Oegopsid sister
Galiteuthis 100% coverage (~11,000 chars), gasbiv OG	Sister of all Deca, excpt Sepia	Sister of all Decabrachia	Onychoteuthis	Oegopsid sister
Ceph only NO OG (Nautilus OG), Architeuthis removed	Sister of all Deca, excpt Sepia	Sister of all Decabrachia	n/a	Oegopsid sister
Ceph only NO OG (Nautilus OG), Chiroteuthis removed	Sister of all Deca, excpt Sepia	Sister of all Decabrachia	Onychoteuthis	Oegopsid sister
Ceph only NO OG (Nautilus OG), Galiteuthis removed	Sister of all Deca, excpt Sepia	Sister of all Decabrachia	Polytomy with Chiroteuthis + Onychoteutis	Oegopsid sister
Ceph only NO OG (Nautilus OG), Onychoteuthis removed	Sister of all Deca, excpt Sepia	Sister of all Decabrachia	Chiroteuthis	Oegopsid sister

Supplementary table 5: alternative topologies and treatments, with key node topology inference. OG = outgroup. SM = supermatrix. Gasbiv = gastropods and bivalves. *Metazoan outgroup* refers to including outgroups to sponges at the base of metazoa.

References

1. Philippe, H., Brinkmann, H., Copley, R. R., Moroz, L. L., Nakano, H., Poustka, A. J., Wallberg, A., Peterson, K. J. & Telford, M. J. 2011 Acoelomorph flatworms are deuterostomes related to Xenoturbella. *Nature* **470**, 255–258.

2. Altschul, S. F., Gish, W., Miller, W., Myers, E. W. & Lipman, D. J. 1990 Basic local alignment search tool. *J. Mol. Biol.* **215**, 403–410.

3. Edgar, R. C. 2004 MUSCLE: multiple sequence alignment with high accuracy and high throughput. *Nucleic Acids Res.* **32**, 1792–1797.

4. Castresana, J. 2000 Selection of conserved blocks from multiple alignments for their use in phylogenetic analysis. *Mol. Biol. Evol.* **17**, 540–552.

5. Vaidya, G., Lohman, D. J. & Meier, R. 2011 SequenceMatrix: concatenation software for the fast assembly of multi-gene datasets with character set and codon information. *Cladistics* **27**, 171–180.

6. Lartillot, N., Rodrigue, N., Stubbs, D. & Richer, J. 2013 PhyloBayes MPI: phylogenetic reconstruction with infinite mixtures of profiles in a parallel environment. *Syst. Biol.* **62**, 611–615.

7. Stamatakis, A. 2014 RAxML version 8: a tool for phylogenetic analysis and post-analysis of large phylogenies. *Bioinformatics* **30**, 1312–1313.

8. Lepage, T., Bryant, D., Philippe, H. & Lartillot, N. 2007 A general comparison of relaxed

molecular clock models. *Mol. Biol. Evol.* **24**, 2669–2680.

9. Thorne, J. L., Kishino, H. & Painter, I. S. 1998 Estimating the rate of evolution of the rate of molecular evolution. *Mol. Biol. Evol.* **15**, 1647–1657.

10. Drummond, A. J., Ho, S. Y. W., Phillips, M. J. & Rambaut, A. 2006 Relaxed phylogenetics and dating with confidence. *PLoS Biol.* **4**, e88.

11. Fedonkin, M. A. & Waggoner, B. M. 1997 The Late Precambrian fossil Kimberella is a mollusc-like bilaterian organism. *Nature* **388**, 868–871.

12. Grabherr, M. G. et al. 2011 Full-length transcriptome assembly from RNA-Seq data without a reference genome. *Nat. Biotechnol.* **29**, 644–652.

13. Guindon, S., Dufayard, J. F., Hordijk, W., Lefort, V. & Gascuel, O. 2009 PhyML: fast and accurate phylogeny reconstruction by maximum likelihood. In *Infection Genetics and Evolution*, pp. 384–385. ELSEVIER SCIENCE BV PO BOX 211, 1000 AE AMSTERDAM, NETHERLANDS.

14. Le, S. Q. & Gascuel, O. 2008 An improved general amino acid replacement matrix. *Mol. Biol. Evol.* **25**, 1307–1320.

15. Lanfear, R., Calcott, B., Ho, S. Y. W. & Guindon, S. 2012 Partitionfinder: combined selection of partitioning schemes and substitution models for phylogenetic analyses. *Mol. Biol. Evol.* **29**, 1695–1701.

16. von Haeseler, A., Minh, B. Q., Nguyen, L. T. & Schmidt, H. A. 2012 IQ-TREE version 0.9. 3 (March 2013) Efficient phylogenetic tree reconstruction and ultrafast bootstrap approximation.

17. Condon, D., Zhu, M., Bowring, S., Wang, W., Yang, A. & Jin, Y. 2005 U-Pb ages from the neoproterozoic Doushantuo Formation, China. *Science* **308**, 95–98.

18. Benton, M. J., Donoghue, P. & Asher, R. J. 2015 Constraints on the timescale of animal evolutionary history. *Palaeontol. Electronica*

19. Nutzel, A. & Bandel, K. 2000 Goniasmidae and Orthonemidae: two new families of the Palaeozoic Caenogastropoda (Mollusca, Gastropoda). *Neues Jahrbuch fur*

20. Parkhaev, P. Y. 2008 The Early Cambrian Radiation of Mollusca. In *Phylogeny and Evolution of the Mollusca* (ed W. Ponder), pp. 33–69. University of California Press.

21. Frýda, J., Nützel, A. & Wagner, P. J. 2008 Paleozoic gastropoda. *Phylogeny and Evolution of the Mollusca*

22. Fuchs, D., Iba, Y., Ifrim, C., Nishimura, T., Kennedy, W. J., Keupp, H., Stinnesbeck, W. & Tanabe, K. 2013 Longibelus gen. nov., a new Cretaceous coleoid genus linking Belemnoidea and early Decabrachia. *Palaeontology* **56**, 1081–1106.

23. Fuchs, D. & Weis, R. 2008 Taxonomy, morphology and phylogeny of Lower Jurassic loligosepiid coleoids (Cephalopoda). *Neues Jahrbuch f??r Geologie und Pal??ontologie - Abhandlungen* **249**, 93–112.

24. Kröger, B. & Mapes, R. H. 2007 On the origin of bactritoids (Cephalopoda).

Paläontologische Zeitschrift **81**, 316–327.

25. Grant, S. W., Knoll, A. H. & Germs, G. J. 1991 Probable calcified metaphytes in the latest Proterozoic Nama Group, Namibia: origin, diagenesis, and implications. *J. Paleontol.* **65**, 1–18.
